# Association of SIRT1 Promoter Polymorphisms with Type 2 Diabetes Mellitus and Pregnancy-Related Complications in the Greek Population

**DOI:** 10.3390/genes16080886

**Published:** 2025-07-27

**Authors:** Sophia Letsiou, Eirini Prountzou, Despina Vougiouklaki, Maria Trapali, Michail Papapanou, Zoe Siateli, Konstantinos Ladias, Dimitra Houhoula, Panagiotis Halvatsiotis

**Affiliations:** 1 Department of Food Science and Technology, Faculty of Food Sciences, University of West Attica, 12243 Athens, Greece; sletsiou@uniwa.gr (S.L.); eirinipro87@hotmail.com (E.P.); dvougiouklaki@uniwa.gr (D.V.); 2 Department of Biomedical Science, University of West Attica, 12243 Athens, Greece; ymaria@uniwa.gr; 3 Second Department of Obstetrics and Gynecology, Aretaieion University Hospital, Medical School, National and Kapodistrian University of Athens, 11528 Athens, Greece; mgpapapanou@gmail.com; 4 Medical School SAPIENZA, Universita di Roma, 00185 Rome, Italy; zoesiateli27@gmail.com; 5 Microbiological Center Life Check, 11526 Athens, Greece; kladias@yahoo.com; 6 2nd Propaedeutic Department of Internal Medicine, Medical School, National and Kapodistrian University of Athens, “ATTIKON” University Hospital, 12461 Chaidari, Greece; pahalv@gmail.com

**Keywords:** type 2 diabetes mellitus, gestational diabetes, preeclampsia, rs12778366, rs3758391, polymorphism, genotype, association study

## Abstract

**Background/Objectives:** SIRT1 is a NAD^+^-dependent protein deacetylase regulating metabolic and stress response pathways. Genetic variations in the *SIRT1* gene may contribute to the pathogenesis of type 2 diabetes mellitus (T2DM). This case–control study investigates the associations of two SIRT1 promoter polymorphisms, rs12778366 and rs3758391, in patients with type 2 diabetes mellitus (T2DM), gestational diabetes mellitus (GDM), preeclampsia, and healthy controls. **Methods:** This case–control study compared the genotypes between T2DM and pregnant and non-pregnant controls. We also compared genotypes between pregnant women with T2DM, GDM, preeclampsia, and healthy pregnant controls. Genomic DNA was extracted and analyzed using PCR-RFLP for the detection of rs12778366 and rs3758391 polymorphisms. Genotype frequencies were compared using chi-square tests, and odds ratios (ORs) with 95% confidence intervals (CIs) were calculated. **Results:** The study included 66 patients with T2DM, 36 with GDM, 12 with preeclampsia, and 81 pregnant and non-pregnant controls (33 pregnant controls). Although rs3758391 was more frequent in T2DM, the difference was not statistically significant between SIRT1 polymorphisms and T2DM. The CT genotype was more prevalent in T2DM (54.5%) compared to controls (33.4%); however, this difference was not significant. We finally found no significant association of the investigated SIRT1 polymorphisms with any of the conditions studied. In addition, the small sample size, especially for preeclampsia cases, limits the statistical power to detect significant associations. **Conclusions:** Although no significant association was observed between SIRT1 polymorphisms and diabetes, the findings of our study underscore the need for further studies examining SIRT1 polymorphisms in various ethnic groups, with a focus on leveraging these genetic variations in diabetes pathophysiology. Larger studies in the Greek population could also provide additional meaningful findings.

## 1. Introduction

Diabetes mellitus (DM) is a complex and heterogeneous group of metabolic disorders characterized by chronic hyperglycemia resulting from defects in insulin secretion, insulin action, or both [1,2]. Among DM clinical forms, type 2 diabetes mellitus (T2DM), as well as gestational diabetes mellitus (GDM), represent two major global public health concerns, particularly considering their increasing prevalence and long-term complications.

Indeed, T2DM represents a significant global public health challenge, currently affecting over 4% of the population, with projections estimating an increase to 5.4% by 2025 [1,2]. Additionally, according to the International Diabetes Federation (IDF), approximately one in two adults living with diabetes worldwide remains undiagnosed. Moreover, the prevalence of prediabetes is largely underestimated, as it is typically identified through either fasting plasma glucose (FPG) measurements or the oral glucose tolerance test (OGTT) [3,4].

On the other hand, GDM is characterized by elevated blood glucose levels first identified during pregnancy [2,3,5]. In the European Region, approximately 9.6% of women aged 25 years and older are affected by GDM [5]. Affecting 3–17% of pregnancies, GDM poses risks such as preterm birth and preeclampsia and increases the likelihood of metabolic and cardiovascular issues for both the mother and offspring. According to a meta-analysis, women with a history of GDM have a sevenfold higher risk of developing T2DM compared to those without prior GDM [6].

In Greece, according to the Hellenic National Nutrition and Health Survey (HNNHS), a representative nationwide study, the prevalence of T2DM in the population was 5.2%, reaching 13.7% in adults aged >60 years (without any significant sex differences) [7]. In addition, the prevalence of GDM reached up to 15% in pregnant women [5,8].

The development of genome-wide association studies (GWAS) improved the understanding of complex diseases like diabetes mellitus by analyzing multiple single-nucleotide polymorphisms (SNPs) across the genome [2]. Genes implicated in DM include *TBC1D4*, *KLF14*, *IRS1*, *KCNJ11*, and *ABCC8*, influencing several processes such as glucose uptake, adipocyte development, and insulin regulation [4,9,10,11,12]. Genes like *TCF7L2*, *GCK*, *KCNJ11*, *KCNQ1*, *CDKAL1*, *MTNR1B*, and *IRS1* are also commonly associated with both GDM and T2DM [13].

The potential association of sirtuins with diabetes has also been extensively examined. Sirtuins, particularly SIRT1, are NAD^+^-dependent deacetylases that regulate various metabolic and physiological processes, including glucose-lipid metabolism, inflammation, autophagy, and circadian rhythm [14,15,16]. Specifically, SIRT1 plays a significant role in enhancing insulin sensitivity by regulating proteins like PGC-1α, FOXO1, PER2, and BMAL1 and influences pathways involving p53, HIFs, NF-κB, and autophagy-related genes [17,18,19,20,21].

Research into polymorphisms of the promoter region of the *SIRT1* gene, such as rs12778366 and rs3758391, has revealed several associations with T2DM, obesity, glucose tolerance, hypertension development risk, and preeclampsia [22,23,24,25,26,27,28]. However, these findings have been inconsistent due to genetic variance among different ethnicities, and data specific to the Greek population, especially among women, remain scarce.

The present study aims to address this knowledge gap by investigating potential associations between the common *SIRT1* promoter polymorphisms, rs12778366 and rs3758391, and T2DM and diabetes-related pregnancy complications in a well-defined cohort of Greek women. By evaluating specific SNPs within the *SIRT1* promoter region, we seek to uncover potential genetic markers of disease susceptibility and understand their possible functional roles in the pathophysiology of T2DM. This investigation could contribute to personalized medicine approaches by identifying at-risk individuals and informing targeted prevention strategies.

## 2. Materials and Methods

### 2.1. Collection of Clinical Samples

We included pregnant patients with T2DM, GDM, preeclampsia, and control groups (healthy pregnant and non-pregnant controls). All samples were then transported in sterile tubes and stored at −70 °C immediately after sampling. Skeletal muscle biopsies were collected from the rectus abdominis muscle during cesarean section procedures and immediately placed in RNAlater buffer (Thermo Fisher Scientific, Bremen, Germany) for stabilization before storage at −70 °C. Whole blood samples were collected in EDTA tubes and similarly stored at −70 °C until processing.

### 2.2. Genomic DNA Extraction

DNA was directly extracted from the skeletal muscle of pregnant women and from total blood of the patients using an automatic extractor with the Whole Blood Nucleic Acid Extraction Kit (Zybio, Chongqing, China), following the protocol recommendations. The purity and the quantity of extracted DNA were evaluated spectrophotometrically by calculating OD260/OD280 (spectrophotometer Epoch, Biotech, Boston, MA, USA). All the samples exhibited 1.8–2 values for the OD260/OD280 ratio. For the PCR reaction, 5 μg of template DNA was used.

### 2.3. Genetic Analysis: Restriction Fragment Length Polymorphism (RFLP)

The target sequences of the two SNPs were multiplied via the polymerase chain reaction (PCR) method using the following primers: for the rs12778366, forward primer 5′-TAAGGCTTCTAGGACTGGAGATGA-3′ (STR1) and reverse primer 5′-GTCCCTTAAGCCTAGTATGGGTTC-3′ (STR2) and for the rs3758391, forward primer 5′-GTCACGCAGGTAATTGATGCAG-3′ (STR3) and reverse primer 5′-GGCTTAGTGGAAAGCCCTTC-3′ (STR4). The primers used in the PCR were forward -5′-TGACTTCAGCTTTACTCTTTGT-3′and reverse -5′-CTGATTGGAAACCTTATTAAG-3′.

PCR was performed in a 50 μL final volume solution using the Master Mix (Hot Start, Promega, Madison, WI, USA). The amplification was conducted by a thermal cycler (96-well thermal cycler, applied Biosystems, Foster City, CA. USA), as follows: an initial denaturation of 10 min at 94 C and a final extension of 10 min at 72 **°**C. The cycle program consisted of a 1 min denaturation at 94 **°**C, 1 min, 35 sec annealing at 55 **°**C, and a 1 min extension at 72 **°**C. The actual length of the PCR products for the rs12778366 polymorphism was 212 bp, while for the rs3758391 polymorphism, it was 241 bp.

For the next step, the PCR products were digested using (rs3758391) the restriction enzymes NlaIII (New England Biolabs, Beverly, MA, USA) at 37 °C for 15 min (1 μL NlaIII, 5 μL buffer, 36 μLH_2_O, and 8 μL PCR product). For the rs12778366 polymorphism, the presence of the C allele resulted in enzymatic digestion, yielding two DNA fragments of 170 bp and 42 bp. In contrast, when the T allele was present, no digestion occurred, and a single 212 bp amplicon was observed. Similarly, for the rs3758391 polymorphism, samples carrying the T allele underwent successful digestion, producing two fragments of 146 bp and 94 bp. Conversely, the presence of the C allele prevented enzymatic cleavage, resulting in an undigested 241 bp PCR product. PCR products, as well as digested PCR products, were separated on a 10% polyacrylamide gel using electrophoresis. All the analysis was performed based on three independent experiments in triplicate for each sample.

### 2.4. Statistical Analysis

Categorical variables were presented as frequencies (%). We compared genotypes among different groups of the study using the chi-square test. We compared genotypes between participants with T2DM and all controls. We also compared genotypes between pregnant women with T2DM, GDM, preeclampsia, and pregnant women without T2DM, GDM, or preeclampsia (i.e., pregnant controls). Odds ratios (ORs) and 95% confidence intervals (CIs) were calculated for binary genotype comparisons. A two-tailed *p*-value less than 0.05 was considered statistically significant. All statistical tests were performed using SPSS (SPSS Inc., Chicago, IL, USA) version 16 software.

## 3. Results

### Genotype Distribution of rs3758391 and rs12778366 Polymorphisms

The study cohort included 66 patients with type 2 diabetes mellitus (T2DM), 36 with gestational diabetes mellitus (GDM), 12 with preeclampsia, and 81 control subjects. Among the controls, 33 were pregnant and served as the reference group for comparisons involving GDM and preeclampsia.

Table 1 summarizes the genotype distributions of the rs12778366 and rs3758391 polymorphisms in patients with T2DM and control subjects.

In the case of rs12778366, the TT genotype was the most prevalent in both groups. Although the TT genotype was more frequent in the T2DM group (77.3%) compared to controls (66.6%), the difference was not statistically significant (χ*^2^* = 1.52, *p* = 0.218; OR = 1.70, 95% CI: 0.81–3.56). Of particular interest is the fact that there were no homozygotes for the rare allele C in our study population. Regarding the frequency of T and C alleles in patients with T2DM and controls (individuals without T2DM), the chi-square test showed that the presence of the C allele was not associated with T2DM (*p*-value = 0.221).

In the case of rs3758391 polymorphisms, we found no significant association between T2DM and genotype (χ*^2^* = 6.82, *p* = 0.088). The CT genotype was more prevalent in T2DM (54.5%) compared to the control group (33.4%). The analysis of the frequency of the two alleles (T and C) showed that the presence of the T allele was not associated with T2DM (*p*-value = 0.225).

Table 2 presents the genotype frequencies of rs3758391 and rs12778366 polymorphisms in the pregnancy-related groups of the study (T2DM, GDM, preeclampsia, and pregnant controls).

In the case of rs12778366 polymorphism, the analysis shows no significant differences in genotypes between pregnant women groups (T2DM, GDM, preeclampsia, and control; χ*^2^* = 4.23, *p* = 0.237; Table 2). Of note, though these differences were non-significant, the TT genotype appeared more frequently in the T2DM and control groups than in preeclampsia cases. Similarly, the rs3758391 genotype distribution among T2DM, GDM, preeclampsia, and control groups was not statistically significant (χ*^2^* = 9.90, *p* = 0.129).

## 4. Discussion

Previous reports have highlighted the potential functional effects of rs12778366 and rs3758391 in the context of SIRT1 expression or promoter activity. Indeed, the rs12778366 (T/C) polymorphism resides within the SIRT1 promoter and has been associated with altered gene regulation. In vitro luciferase reporter assays and haplotype analyses suggest that when the C allele is present (particularly in combination with the wild-type background), promoter activity is significantly reduced—by over 50% relative to the all-wild-type haplotype—implying diminished SIRT1 transcriptional activation [23]. Clinical studies further support these functional implications: carriers of the C allele (i.e., TC or CC) have been linked to higher mortality under elevated IL-6 conditions and poorer glucose tolerance, potentially reflecting lower SIRT1 levels controlling metabolism or inflammation.

Conversely, rs3758391 (T/C) lies near a p53 binding site in the promoter and has been experimentally shown to modulate SIRT1 induction under nutrient stress. In human skeletal muscle, individuals harboring the T allele exhibit greater upregulation of SIRT1 mRNA in response to calorie restriction or combined calorie restriction plus exercise, paralleling enhanced expression of downstream targets such as AMPKα2 and PGC-1β [29]. Clinically, the T allele in SLE patients correlates with increased disease severity and renal involvement, potentially driven by overexpression of SIRT1 in immune cells [30]. Taken together, these data suggest a regulatory dichotomy: rs12778366-C may repress basal promoter/enhancer activity and diminish SIRT1 expression, while rs3758391-T enhances SIRT1 inducibility under stress or in specific tissues, thereby influencing metabolic, inflammatory, and aging-related phenotypes.

In this case–control study, we compared genotype frequencies of rs3758391 and rs12778366 polymorphisms between pregnant women with T2DM and 81 healthy pregnant and non-pregnant controls, and the frequencies of the same polymorphisms between pregnant women with T2DM, GDM, pre-eclampsia, and 33 pregnant healthy controls (a subgroup of the 81 overall control group) to clarify in more detail the relation among rs3758391 and rs12778366 polymorphisms with T2DM in the Greek population.

Although our findings may suggest an increased probability of rs3758391 in those with T2DM compared to controls, this association was not significant. The significant enrichment of the CT genotype in the T2DM group could reflect a functional impact of this genotype in metabolic regulation or inflammation pathways; however, this remains unclear. A study by Kaabi et al. (2024) reported no significant association of rs3758391 with T2DM in a Saudi Arabian population, while Ahmed et al. (2025) reported that the rs3758391 TT genotype increased the risk of T2DM in a Bangladeshi population [31,32].

Cruz et al. (2010) linked the T allele of rs3758391 to T2DM in a Mexican population [33]. Similarly, Fardonbeh et al. (2020) observed a strong association between the same allele and T2DM in the Iranian population [34]. In contrast, studies by Peng et al. (2018) and Kovanen et al. (2015) did not find a significant link between rs3758391 and T2DM in Chinese and Finnish populations, respectively [25,35]. Interestingly, Sadeghi et al. (2021) reported that the SIRT1 variant, rs3758391, might play a protective role against T2DM in the Iranian population [24]. Similarly, Botden et al. (2012) demonstrated that SIRT1 variants, particularly in interaction with prenatal famine exposure, may influence T2DM susceptibility, suggesting a gene–environment interaction that may mitigate risk [36]. Furthermore, Dong et al. (2011) reported that specific upstream polymorphisms of SIRT1 (e.g., rs10509291) were associated with reduced insulin secretion and a lower T2DM risk in Pima Indians, indicating that some SIRT1 polymorphisms may modify diabetes risk via effects on β-cell function [37]. These conflicting findings could be partly due to ethnic differences, as genetic variations may influence the expression levels of the SIRT1 protein differently across populations, thereby affecting susceptibility to T2DM either positively or negatively. These studies support our findings and may suggest the potential role of rs3758391 in diabetes-related pathways.

The lack of significant findings for rs12778366 in our study might be attributed to the limited sample size. However, this outcome is consistent with the study by Kaabi et al. (2024), which reported no significant association of rs12778366 with T2DM in a Saudi Arabian population [31], as well as with Kovanen et al. (2015), who did not find a significant link between rs12778366 and T2DM in Finnish populations [35]. However, a study by Rai et al. reported that the TT genotype of rs12778366 was positively correlated with T2DM risk in an Indian population [23], and Sadeghi et al. (2021) reported a positive correlation of the C allele of the same variant with T2DM in an Iranian population [24]. Interestingly, other SIRT 1 polymorphisms, such as rs3740051 and rs35995735, were associated with T2DM risk [38]. In a Dutch population, SIRT1 gene SNPs are associated with prenatal famine exposure, influencing the T2D risk [34]. In Pima Indians, an upstream variant rs10509291, as well as an intron variant rs7896005 in the SIRT1 gene, were correlated with reduced insulin secretion and increased risk of T2D [35].

Among pregnant women, neither polymorphism showed a significant association with GDM or preeclampsia. To the best of our knowledge, there are no studies investigating the associations of rs12778366 and rs3758391 polymorphisms with T2DM, GDM, and preeclampsia among pregnant women. A study by Dimitrenko et al. (2025) reported a significant association between the rs7895833 AG genotype and GDM and preeclampsia in a Russian population [39].

Several recent studies have investigated the association between promoter polymorphisms of the SIRT1 gene, primarily rs12778366, rs3758391, and rs7895833, and their relationship with T2DM and pregnancy complications (Table 3). While T2DM has been the primary focus, only a limited number of studies have examined potential associations with GDM (Table 3). Among these variants, rs12778366 and rs3758391 are the most frequently studied (Table 3).

These discrepancies may reflect ethnic genetic variability, environmental or lifestyle influences, and variation in study design and power. It is also possible that distinct genetic mechanisms contribute to T2DM and GDM across populations. According to Table 3, SIRT1 promoter polymorphisms appear to influence T2DM risk in certain populations, especially in Asia, but do not consistently demonstrate the same effect in European or Middle Eastern cohorts [24,31,32,34,38,39]. In addition, the rs3758391 variant shows the most robust association with T2DM outside Europe, while rs12778366 may play a regulatory role in gene expression rather than acting as an independent risk marker. The rs7895833 polymorphism may be a promising genetic marker for GDM, particularly in women of reproductive age. These findings emphasize the need for larger, ethnically diverse studies to clarify the clinical relevance of SIRT1 promoter variants in metabolic disorders. In addition, the link between GDM and SIRT1 promoter variants needs to be explored further.

To the best of our knowledge, this is the first study to compare the rs12778366 and rs3758391 SIRT1 promoter polymorphisms between T2DM and pregnant and non-pregnant controls in a Greek population. The study also compared genotypes between pregnant women with T2DM, GDM, preeclampsia, and healthy pregnant controls. However, the study has certain limitations. First, it is a case–control study; hence, it is subject to selection and confounding biases associated with the observational case–control design. The study may have also been underpowered to detect smaller effects due to the limited sample size, particularly in the case of preeclampsia participants. In addition, PCR-RFLP, even though a well-established method, may possess some limitations as it may lead to false genotyping results due to a lack of sensitivity and specificity compared to more advanced techniques such as sequencing or real-time PCR. Therefore, larger studies, even in the Greek population, are required to reach more robust conclusions. Finally, the study included a Greek population of mostly pregnant women. Our findings are limited by sample size and population homogeneity, warranting further studies in diverse cohorts.

## 5. Conclusions

Although the TC genotype of the rs3758391 polymorphism was increased in the T2DM group, no significant associations of rs3758391 and rs12778366 with T2DM or any related pregnancy complication (preeclampsia or GDM) were observed. Our findings highlight the importance of considering the “genotype mosaic” due to genetic variations in different ethnicities and how these variations interact with other genes involved in the same pathway. We suggest that multiple layers of genotypes, both from known and yet-to-be-identified genes, may contribute to the biological mechanisms underlying T2DM. The expression of SIRT1 may be affected by additional functional variants within the gene. Therefore, a comprehensive investigation of SIRT1 gene variations in the Greek population, along with expanding this study to a larger dataset, is necessary to draw more definitive conclusions.

## Figures and Tables

**Table 1:** Genotype distributions of rs12778366 and rs3758391 in type 2 diabetes mellitus (T2DM) cases and controls of the study.

rs12778366					
Group	Genotype	Count (n)	Percentage (%)	Chi^2^/*p*-Value	OR (95% CI)/Power
T2DM (n = 66)	TT	51	77.3	1.52/0.218 *^,1^	1.70 (0.81–3.56)/29.4%
	TC	15	22.7		
Control (n = 81)	TT	54	66.6		
	TC	27	33.4		
rs3758391					
Group	Genotype	Count (n)	Percentage (%)	Chi^2^/*p*-Value	OR (95% CI)/Power
T2DM (n = 66)	CC	24	36.3	6.82/0.088 *^,2^	74.2%
	CT	36	54.5		
	TT	6	9.1		
Control (n = 81)	CC	45	55.5		
	CT	27	33.4		
	TT	9	11.1		

* Statistical analysis performed using Pearson’s Chi-square test. ^1^ T2DM vs. control, TT vs. TC, ^2^ T2DM vs. control, CC vs. CT vs. TT.

**Table 2:** Genotype distribution of rs3758391 and rs12778366 in the subgroup of pregnant women of the study (T2DM, GDM, preeclampsia, and control).

rs12778366					
Group	Genotype	Count (n)	Percentage (%)	Chi^2^/*p*-Value	OR (95% CI)/Power
T2DM (n = 66)	TT	51	77.3	4.23/0.237 *^,1^	53.9%
	TC	15	22.7		
GDM (n = 36)	TT	24	66.6		
	TC	12	33.4		
Preeclampsia (n = 12)	TT	6	50		
	TC	6	50		
Control (n = 33)	TT	24	72.8		
	TC	9	27.2		
rs3758391					
Group	Genotype	Count (n)	Percentage (%)	Chi^2^/*p*-Value	OR (95% CI)/Power
T2DM (n = 66)	CC	24	36.3	9.90/0.129 *^,2^	88.2%
	CT	36	54.5		
	TT	6	9.1		
GDM (n = 36)	CC	21	58.3		
	CT	12	33.4		
	TT	3	8.3		
Preeclampsia (n = 12)	CC	6	50		
	CT	3	25		
	TT	3	25		
Control (n = 33)	CC	18	54.5		
	CT	12	36.4		
	TT	3	9.1		

* Statistical analysis performed using Pearson’s Chi-square test. ^1^ T2DM vs. control, CC vs. CT vs. TT, ^2^ T2DM vs. GDM vs. preeclampsia vs. control, TT vs. TC.

**Table 3:** Studies on SIRT1 promoter polymorphisms and their association with T2DM and pregnancy complications in different ethnic groups.

Study Location	Polymorphism(s)	Condition	Population Details	Key Findings	References
Greece (Current Study)	rs12778366, rs3758391	T2DM, GDM, preeclampsia	66 patients with T2DM, 36 with GDM, 12 with preeclampsia, and 112 matched controls	No statistically significant association found	Current Study (2025)
Saudi Arabia	rs12778366, rs3758391	T2DM	221 patients, 224 controls	Genotype frequencies were similar between T2DM and controls; no significant association	Kaabi et al., 2024 [31]
China	rs3740051, rs35995735	T2DM	218 patients, 358 controls	Significant association (ORs 1.75 and 3.58), relevant haplotype risk	Pang et al., 2023 [38]
Russia	rs12778366, rs7895833	GDM, preeclampsia	61 patients with GDM and PE, 63 patients with GDM (controls)	The rs12778366 is associated with shorter telomeres and an increased risk of developing PE.	Dmitrenko et al., 2025 [39]
Bangladesh	rs3758391	T2DM	72 T2DM patients, 90 healthy controls	T allele associated with increased T2DM risk (OR = 3.88, *p* = 0.012)	Ahmed et al., 2025 [32]
Iran	rs12778366, rs3758391	T2DM	403 patients with T2DM and 410 healthy controls	The C allele of rs12778366 and the T allele of rs3758391 were linked to an increased risk of T2DM	Sadeghi et al., 2021 [24]
Southwestern Iran	rs3758391	T2DM	132 patients with T2DM with or without nephropathy	The TT genotype and the T allele carrier of rs3758391 were strongly associated with T2DM	Faradonbeh et al., 2020 [34]

## Data Availability

The original contributions presented in this study are included in the article. The data of the current study are available from the corresponding author upon reasonable request.
